# Sponge Grafting

**Published:** 1894-06

**Authors:** S. A. Pancoast

**Affiliations:** Ashtabula, Ohio


### Domestic Correspondence.

### SPONGE GRAFTING.

ASHTABULA, OHIO, April 14, 1894.

TO THE EDITOR:

SIR,—In answer to Dr. M. L. Rhein, page 249, April INTERNATIONAL DENTAL JOURNAL, "How shall we restore lost tissue?"

dissect away gum, remove all deposits and affected bone, replace lost process with sterilized (in an oven) sponge, close wound, steady the teeth; nature does the rest. Sponge acts as nucleus for the bone pabulum, and, being an animal substance, is absorbed and carried off through the circulation.

S. A. Pancoast, D.D.S.

